# Dapsone-Induced Methemoglobinemia in a Patient With Multiple Myeloma Following Autologous Stem Cell Transplantation: A Case of Conservative Management

**DOI:** 10.7759/cureus.96903

**Published:** 2025-11-15

**Authors:** Supipi Weragoda, Shreeya Shakya

**Affiliations:** 1 Internal Medicine, Maidstone and Tunbridge Wells NHS Trust, Royal Tunbridge Wells, GBR

**Keywords:** autologous stem cell transplant, dapsone, methemoglobinemia, multiple myeloma, pneumocystis jirovecii pneumonia

## Abstract

Dapsone is an alternative medication for the prophylaxis of *Pneumocystis jirovecii* pneumonia (PJP) in immunocompromised patients. One potential complication associated with dapsone use is acquired methemoglobinemia. However, cases of methemoglobinemia in patients with IgG kappa myeloma following autologous stem cell transplant are rare.

We describe the case of a 67-year-old woman with IgG kappa myeloma who underwent a stem cell transplant and was participating in the RADAR Myeloma XV trial. She received dapsone for PJP prophylaxis due to an allergy to trimethoprim-sulfamethoxazole (TMP-SMX). Two weeks after starting dapsone, she presented with shortness of breath and an oxygen saturation level of 89% on room air. Her arterial blood gas analysis revealed a methemoglobin level of 10.9%. Conservative management, involving the discontinuation of dapsone and supplemental oxygen, led to clinical improvement.

This case highlights the importance of maintaining vigilance for acquired methemoglobinemia, particularly in immunocompromised patients presenting with unexplained hypoxia. Prompt recognition and discontinuation of dapsone are essential for effective management. In this instance, conservative treatment proved successful at a methemoglobin level of 10.9%. This case contributes to the limited evidence supporting the efficacy of conservative management in dapsone-induced methemoglobinemia among post-transplant patients.

## Introduction

Multiple myeloma is a common hematological malignancy characterized by clonal proliferation of plasma cells and multi-organ dysfunction. The RADAR Myeloma XV trial is a UK-based study for newly diagnosed myeloma patients eligible for high-dose therapy and stem cell transplantation [1]. In this setting, post-transplant infection prophylaxis is an important component of patient management, particularly for patients undergoing high-dose therapy and stem cell transplantation, who are at increased risk of opportunistic infections.

We present a rare instance of post-transplant methemoglobinemia in a patient receiving dapsone for *Pneumocystis jirovecii* pneumonia (PJP) prophylaxis due to allergy to trimethoprim-sulfamethoxazole (TMP-SMX), which was successfully managed without antidotal therapy.

Following stem cell transplantation, patients frequently develop nonspecific symptoms such as fatigue, dyspnea, and cyanosis, which may result from anemia, infection, pulmonary complications, or drug toxicity. Drug-induced cases account for the majority of acquired methemoglobinemia, with dapsone being a common cause [2,3]. Widely used for PJP prophylaxis in immunocompromised patients, dapsone is metabolized to hydroxylamine derivatives that directly oxidize hemoglobin [4].

Most reported cases of dapsone-induced methemoglobinemia are symptomatic and require methylene blue or exchange transfusion. Milder cases may be clinically silent and resolve with conservative measures, although such reports in post-transplant populations remain rare [5].

## Case presentation

A 67-year-old woman with a history of IgG kappa multiple myeloma presented to the emergency department with a one-week history of shortness of breath and a general feeling of unwellness. She had been diagnosed with myeloma one year earlier, following a thoracic vertebral fracture, with bone marrow biopsy confirming the diagnosis and Revised ISS Stage II disease. She was recruited into the RADAR Myeloma XV trial and received four cycles of induction therapy with lenalidomide, bortezomib, dexamethasone, and cyclophosphamide. Five months before presentation, she underwent high-dose melphalan conditioning followed by an autologous stem cell transplant, which was complicated by mucositis, reduced appetite, weight loss, and refeeding syndrome.

Post-transplant, the patient commenced maintenance therapy with isatuximab. Two weeks before admission, she was started on dapsone 100 mg daily for PJP prophylaxis due to a TMP-SMX allergy. Her regular medications also included omeprazole, acyclovir, allopurinol, calcium, and vitamin D supplements.

On examination, she appeared alert and oriented, with no visible cyanosis. Heart sounds were normal, and breath sounds were clear bilaterally without crackles or wheeze. There was no peripheral edema, her abdomen was soft and non-tender, and neurological examination was unremarkable. Vital signs included a temperature of 37°C, a heart rate of 96 bpm, blood pressure of 131/70 mmHg, a respiratory rate of 18/min, and an oxygen saturation of 89% on room air, improving to 92%-94% on 4 L of oxygen via nasal prongs.

Laboratory and arterial blood gas (ABG) results are summarized in Table 1, showing normal inflammatory and biochemical parameters. A “saturation gap” was noted between pulse oximetry and arterial oxygen measurements, with a methemoglobin level of 10.9%.

**Table 1:** Laboratory blood investigations and initial arterial blood gas analysis

Laboratory parameters	Patient value	Reference range
WBC	6.37 × 10⁹/L	4.00-10.00 × 10⁹/L
Neutrophils	4.11 × 10⁹/L	2.00-7.00 × 10⁹/L
Hemoglobin	114 g/L	120-150 g/L
Platelets	213 × 10⁹/L	150-410 × 10⁹/L
CRP	3 mg/L	<5 mg/L
Sodium	139 mmol/L	133-146 mmol/L
Potassium	3.5 mmol/L	3.5-5.3 mmol/L
Bilirubin	15 µmol/L	<21 µmol/L
ALP	64 U/L	30-130 U/L
ALT	20 U/L	<35 U/L
ABG	-	-
pH	7.45	7.35-7.45
PaCO_2_	3.9 kPa	4.6-6.4 kPa
PaO_2_	13.3 kPa	11.4-14.4 kPa
HCO_3_	21.9 mmol/L	21-28 mmol/L
Base excess	-2.4	-2 to +2 mmol/L
MetHb	10.9%	0.0%-1.5%

Chest X-ray was normal, CT pulmonary angiography excluded pulmonary embolism and confirmed collapse of the T6 vertebral body, and ECG demonstrated sinus rhythm. Normal hemoglobin levels and inflammatory markers excluded anemia and infection, while imaging ruled out pulmonary complications such as embolism or pneumonia.

In view of the methemoglobin level of 10.9% and clinical stability, conservative management was adopted. Dapsone was discontinued immediately, and the patient received supplemental oxygen and close monitoring. Methylene blue was not administered as the level was below the threshold for antidotal therapy.

**Figure 1:**
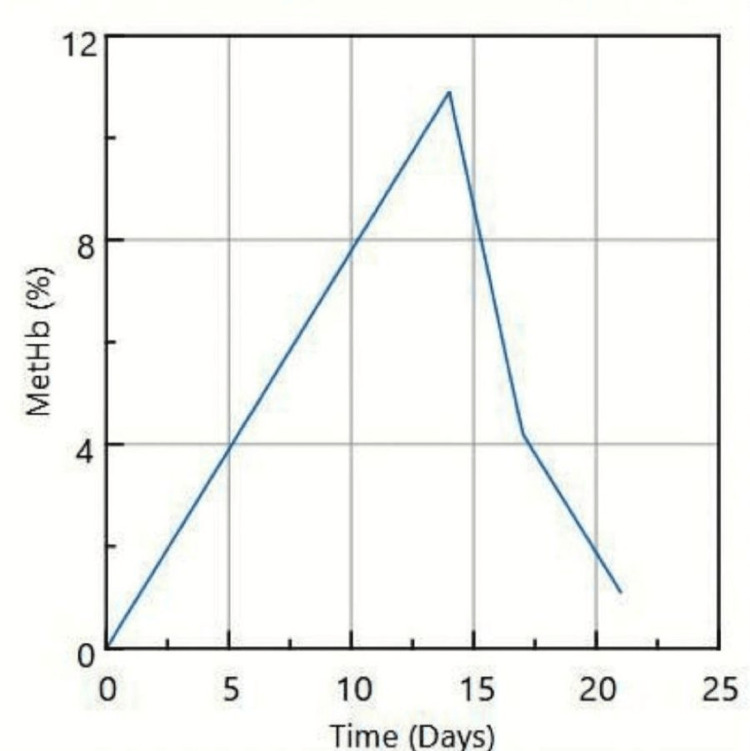
Trend of methemoglobin (MetHb) levels following dapsone initiation MetHb levels decreased from 10.9% at presentation (day 14 after dapsone initiation) to 4.2% on day 17 and normalized to 1.1% by day 21 with conservative management.

Follow-up arterial blood gases indicated a reduction in methemoglobin levels, decreasing from 10.9% at presentation to 4.2% after three days of dapsone withdrawal, and further down to 1.1% after seven days (Figure 1).

Her symptoms steadily improved over the following days, with her oxygen saturation improving to 96%. She was subsequently discharged in stable condition.

## Discussion

This case highlights a rare episode of dapsone-induced methemoglobinemia in a patient with IgG kappa myeloma following autologous stem cell transplantation, who received the medication for PJP prophylaxis. Dapsone is widely used when TMP-SMX is contraindicated, but it can cause oxidative complications, including hemolysis and methemoglobinemia [6].

Methemoglobinemia results from oxidative stress that converts hemoglobin iron from the ferrous (Fe²⁺) to the ferric (Fe³⁺) state, producing methemoglobin, which cannot effectively bind or release oxygen [7]. This oxidation reduces the blood’s oxygen-carrying capacity and shifts the oxygen dissociation curve to the left, impairing tissue oxygen delivery. Under physiological conditions, methemoglobin levels are maintained below 1% through the action of NADH-dependent cytochrome b5 reductase, which reduces methemoglobin back to functional hemoglobin [8]. When oxidative stress exceeds the capacity of this enzymatic pathway, methemoglobin accumulates, leading to toxic levels.

Dapsone toxicity, particularly methemoglobin formation, results from N-oxidation mediated by cytochrome P450 enzymes, producing hydroxylamine metabolites. CYP2E1 is the primary isoenzyme responsible for this reaction in vivo, generating oxidative stress that predisposes susceptible individuals to methemoglobinemia [9]. 

Glucose-6-phosphate dehydrogenase (G6PD) deficiency affects over 400 million people globally and reduces antioxidant capacity. It increases vulnerability to hemolysis and methemoglobinemia with oxidant drugs, including dapsone [10]. In post-transplant patients, pharmacogenetic factors, along with concurrent medications and altered metabolism, increase the risk of methemoglobinemia. This highlights the need for individualized pharmacovigilance and careful selection of agents for PJP prophylaxis.

**Figure 2:**
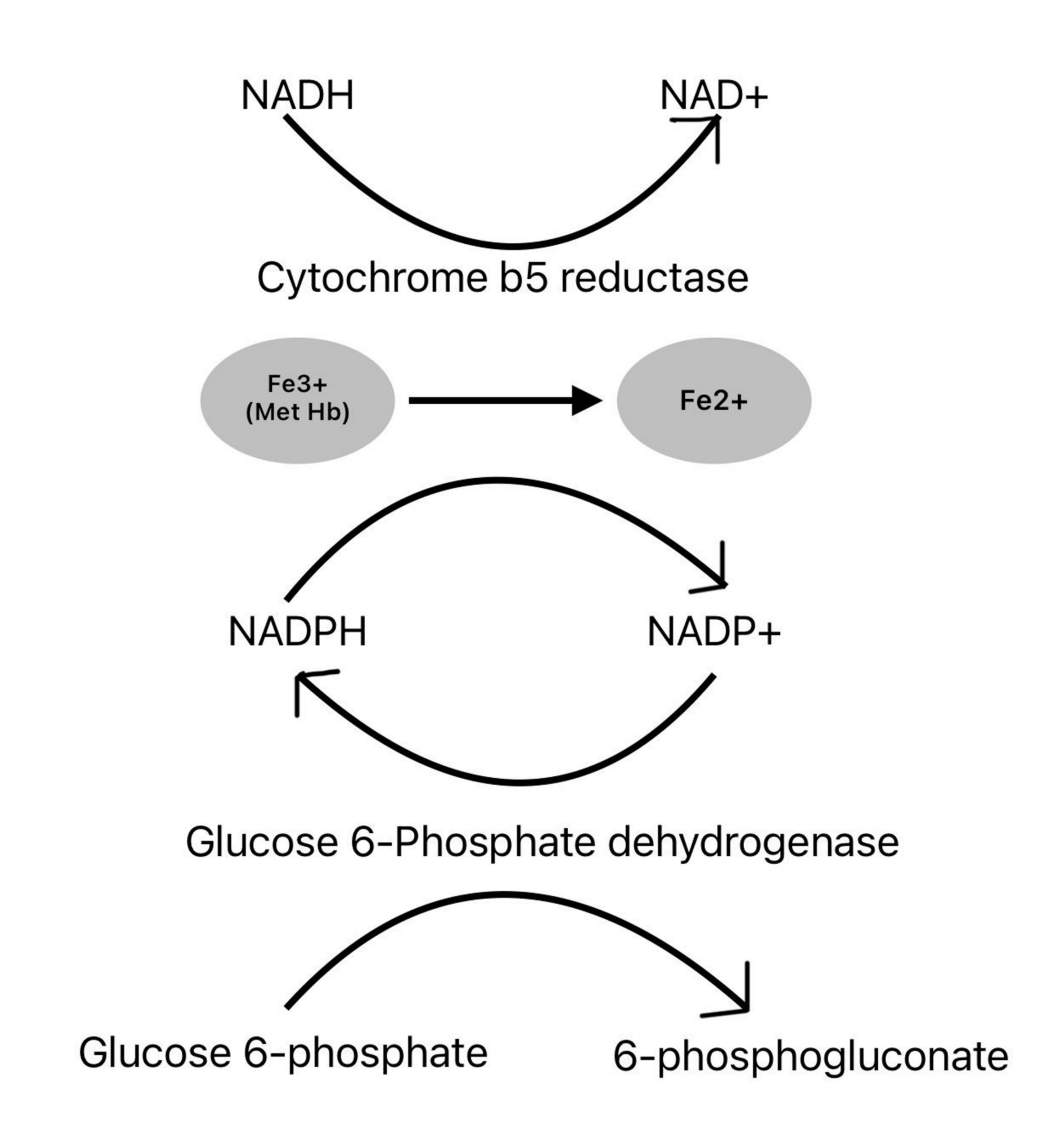
Enzymatic pathways responsible for reducing methemoglobin to functional hemoglobin This figure illustrates the enzymatic pathways, including cytochrome b5 reductase, that reduce methemoglobin to functional hemoglobin, and how enzymatic deficiencies can lead to methemoglobinemia. Image Credit: Author's original creation.

Figure 2 illustrates the enzymatic pathways that reduce methemoglobin to functional hemoglobin.

**Figure 3:**
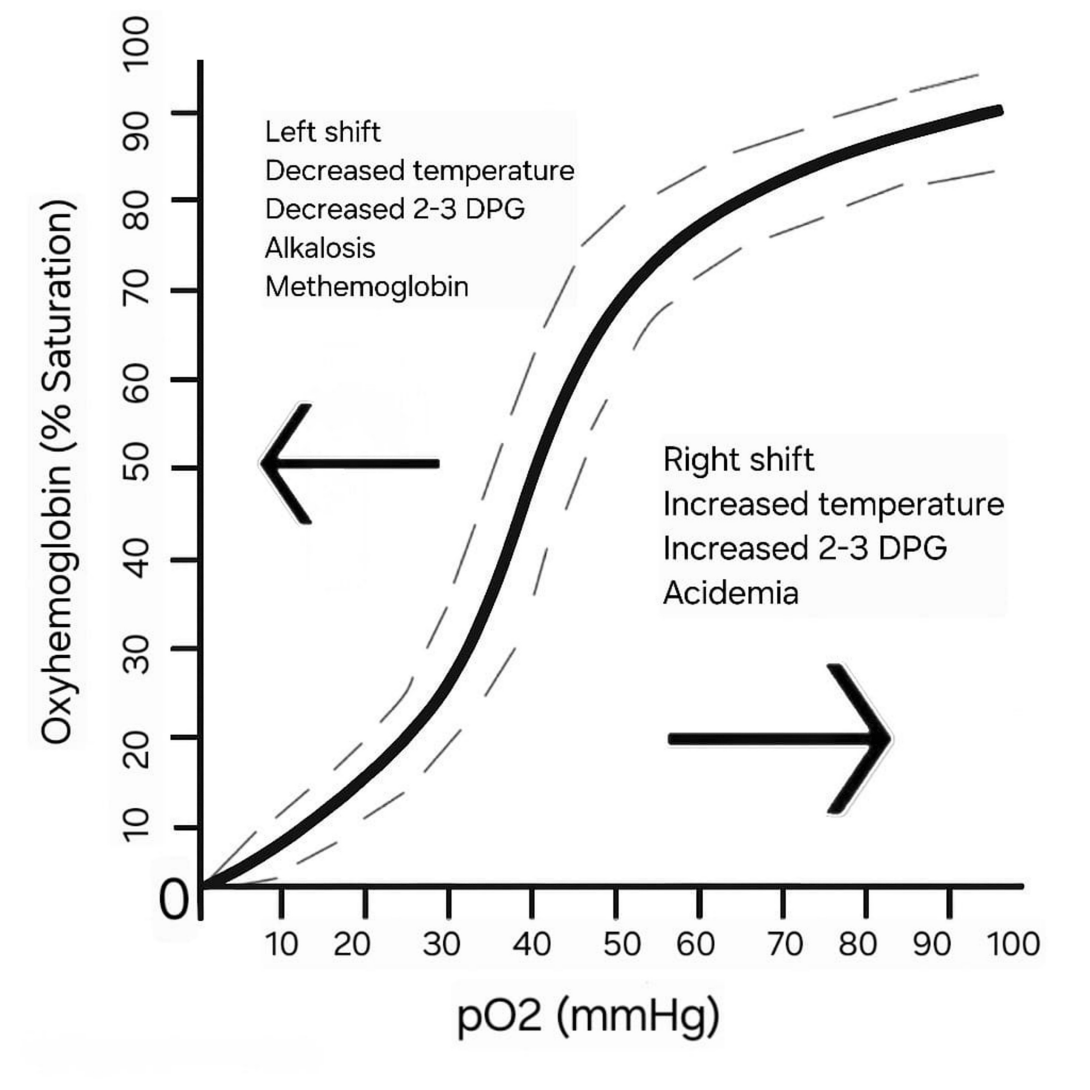
Left shift of oxyhemoglobin dissociation curve in methemoglobinemia This figure demonstrates how methemoglobinemia impairs oxygen release to tissues, explaining hypoxia despite normal arterial oxygen levels. Image Credit: Author's original creation.

 Figure 3 demonstrates how methemoglobinemia impairs oxygen release to tissues.

Stem cell transplant recipients may be especially vulnerable due to polypharmacy with interacting agents, altered hepatic metabolism, and limited hematologic reserve. Although symptomatic cases are relatively uncommon, several reports describe early toxicity, even at standard prophylactic doses [11].

Clinically, methemoglobinemia often presents with non-specific symptoms such as cyanosis, dyspnea, and fatigue. A diagnostic hallmark is the “saturation gap,” where oxygen saturation is low on pulse oximetry despite a relatively normal arterial oxygen tension [8]. Recognition can be challenging in immunocompromised patients, in whom hypoxia is often attributed to infection or cardiopulmonary disease [2,3].

The management of methemoglobinemia varies based on the severity of the patient's condition. For asymptomatic patients or those with methemoglobin levels below 20%, simple measures, such as discontinuing the causative agent, may be sufficient. However, for symptomatic patients or those with methemoglobin levels exceeding 20%, treatment with methylene blue is recommended [12,13]. Methylene blue accelerates the reduction of methemoglobin but is contraindicated in G6PD deficiency due to the risk of hemolysis. Exchange transfusion or hyperbaric oxygen may be considered for refractory or life-threatening cases.

In our patient, the methemoglobin level peaked at 10.9% and resolved with cessation of dapsone alone, supporting conservative management without the need for methylene blue. Ongoing quantitative monitoring ensured clinical stability and safe recovery. This case underscores the importance of considering methemoglobinemia in post-transplant patients presenting with unexplained hypoxia. Awareness of this potential complication and early recognition can prevent morbidity and allow timely modification of prophylactic strategies.

## Conclusions

Dapsone remains a useful alternative for PJP prophylaxis in patients who cannot tolerate TMP-SMX, but clinicians must remain alert to the risk of methemoglobinemia, even at prophylactic doses. Patients with hematological malignancies and those who have undergone stem cell transplantation may be particularly susceptible due to altered metabolism, comorbidities, and polypharmacy.

This case highlights a rare but important presentation of dapsone-induced methemoglobinemia in a patient with multiple myeloma five months post-autologous stem cell transplantation while enrolled in the RADAR Myeloma XV trial. The patient developed mild methemoglobinemia (10.9%) after two weeks of therapy, which resolved with conservative management following drug withdrawal alone. This case contributes to the limited evidence supporting the success of conservative management of dapsone-induced mild methemoglobinemia in post-transplant patients. Routine methemoglobin monitoring should be considered for post-transplant patients newly started on dapsone. Early recognition and appropriate management can prevent severe complications.
